# Three-Dimensional Dynamic Analyses of Track-Embankment-Ground System Subjected to High Speed Train Loads

**DOI:** 10.1155/2014/924592

**Published:** 2014-02-26

**Authors:** Qiang Fu, Changjie Zheng

**Affiliations:** Key Laboratory of Ministry of Education for Geomechanics and Embankment Engineering, Hohai University, Nanjing 210098, China

## Abstract

A three-dimensional finite element model was developed to investigate dynamic response of track-embankment-ground system subjected to moving loads caused by high speed trains. The track-embankment-ground systems such as the sleepers, the ballast, the embankment, and the ground are represented by 8-noded solid elements. The infinite elements are used to represent the infinite boundary condition to absorb vibration waves induced by the passing of train load at the boundary. The loads were applied on the rails directly to simulate the real moving loads of trains. The effects of train speed on dynamic response of the system are considered. The effect of material parameters, especially the modulus changes of ballast and embankment, is taken into account to demonstrate the effectiveness of strengthening the ballast, embankment, and ground for mitigating system vibration in detail. The numerical results show that the model is reliable for predicting the amplitude of vibrations produced in the track-embankment-ground system by high-speed trains. Stiffening of fill under the embankment can reduce the vibration level, on the other hand, it can be realized by installing a concrete slab under the embankment. The influence of axle load on the vibration of the system is obviously lower than that of train speed.

## 1. Introduction

In recent years, with the rapid development of high speed railway in China, the issue on dynamic response of soil and environment vibration induced by the moving trains has been paid more attention, and the researchers have paid attention to the dynamic effects associated with train-track interaction [1–8]. A significant trendy in this context is the high speed and self-weight of railway trains, which impose heavier loads on the tracks [9]. The moving high speed train often produces significant ground vibrations, especially at the resonance condition. Thus, how to avoid resonance and reduce the vibrations has become a key researching issue.

The soil vibration problem was first studied by Lamb in 1904, whose pioneering research mainly focused on the dynamic response of elastic half-space and elastic body with infinite boundary generated by stationary loads or approximately stationary loads [9]. The moving-load effects have been studied for beam structures and beams on Winkler foundation [10, 11]. A prediction model developed by Krylov [12] was used [13, 14]. The steady-state vibration of a periodically supported beam on an elastic half-space under a uniformly moving harmonic load has been studied in [15]. Krylov's model is valid when the train speed near the critical phase velocity of the coupled track-soil system, as the quasi-static excitation, is dominant in this case [16–18]. Sheng et al. [19] studied the ground vibration generated by a harmonic load that moves along a railway track. Kaynia et al. [2], along with their presentation of measurements, presented the development of an FEM model composed of a moving load on a railway/embankment structure, which was coupled to a layered ground model at a series of points via the Green's functions. Kargarnovin et al. [20] have studied the response of infinite beams supported by nonlinear viscoelastic foundations subjected to harmonic moving loads. In [21, 22], a closed-form displacement response of beams on viscoelastic foundations has been presented for two cases of a line moving load and a concentrated moving load. Takemiya and Bian [23] and Picoux and Le Houédec [24] studied the dynamic responses of a track system on the ground subjected to a moving train load. In [25, 26], an analytical approach was used to investigate dynamic responses of a track system and the poroelastic half-space soil medium subjected to a moving point load under three-dimensional condition. In [27], Cao et al. investigated the vibrations of railway tracks on a poroelastic half-space generated by moving trains through a vehicle-track-ground coupling model. In [28], the dynamic response of a fully saturated poroelastic half-space due to accelerating or decelerating trains is investigated by a semianalytical method. In [29], the proposed approach was verified by the semianalytical solutions for a 3D saturated half space subjected to a moving load. Sheng et al. [30] also use a FEM/BEM approach for the analysis of ground vibration produced by trains. Galvín and Domínguez [31, 32] presented a general and fully three-dimensional model for analysis of the soil motion and the effects of HST (high speed train) passage on nearby surface and underground structures. In [33], a general and fully three-dimensional multibody finite element boundary element model, formulated in the time domain to predict vibrations due to train passage at the vehicle, the track, and the free field, is presented. Kaynia et al. [2] and Takemiya and Bian [23] have done some works specifically to railway embankments by using the boundary element method. Hall [34] and Ju et al. [35] used the finite element method to perform three-dimensional time domain simulation for train-induced vibrations of embankment on layered ground. The rails, sleepers, ballast, embankment, and layered ground were all described in detail. The elastically distributed wheel load has been considered by Krylov et al. [36, 37] and Takemiya [38]. However, they have not proceeded to investigate the vehicles traveling effect under various speeds. By using Green's functions for a layered half-space, or combined with a BEM FEM method, different authors have proposed approaches for analyzing soil induced vibrations due to moving loads. Most of the existing papers discussed the track-soil interaction problem in the frequency wave number domain by using Fourier transformation. The moving point loads were applied on the nodes in the beam elements for simulating the rail. However, the dynamic response of rack-embankment-ground system cannot be obtained in time domain with simulation accurately. For three-dimensional finite element models, the railway track is represented by 8-noded solid elements, the dynamic response can be obtained in the simulation. The validation of the simulation model is important. Kaewunruen and Remennikov [39] have dealt with the application of vibration measurements and finite element model updating to the assessment of ballasted rail track sleepers and indicated that the model proved its effectiveness for predicting the free vibration characteristics of *in situ* sleepers under different circumstances. In [40], the finite element model of the railway concrete sleeper was previously established and validated against experimental data by the authors.

In this research, the dynamic three-dimensional (3D) finite element program ABAQUS was chosen for creating the models used to simulate the vibration of track-embankment-ground system induced by high speed train moving on the track system. The elements on the rail top are referred to as loading unit, and the moving loads were applied on the loading unit directly to simulate the train moving load. Four train speeds (60, 80, 100, and 120 m/s, resp.) were considered. These speeds were smaller, closer, or greater than the Rayleigh wave speed of the ground. The three-dimensional model analyses are presented in both the time and frequency domains. The effect of material parameters, especially the modulus changes of ballast, embankment, fill, and soil on the dynamic response of track system was taken into account. The ground vibration characteristics of track-ballast-embankment-ground system are analyzed under different train moving speeds.

## 2. Numerical Model of Track-Embankment-Ground System

### 2.1. Train Load Model

**Figure 1:**
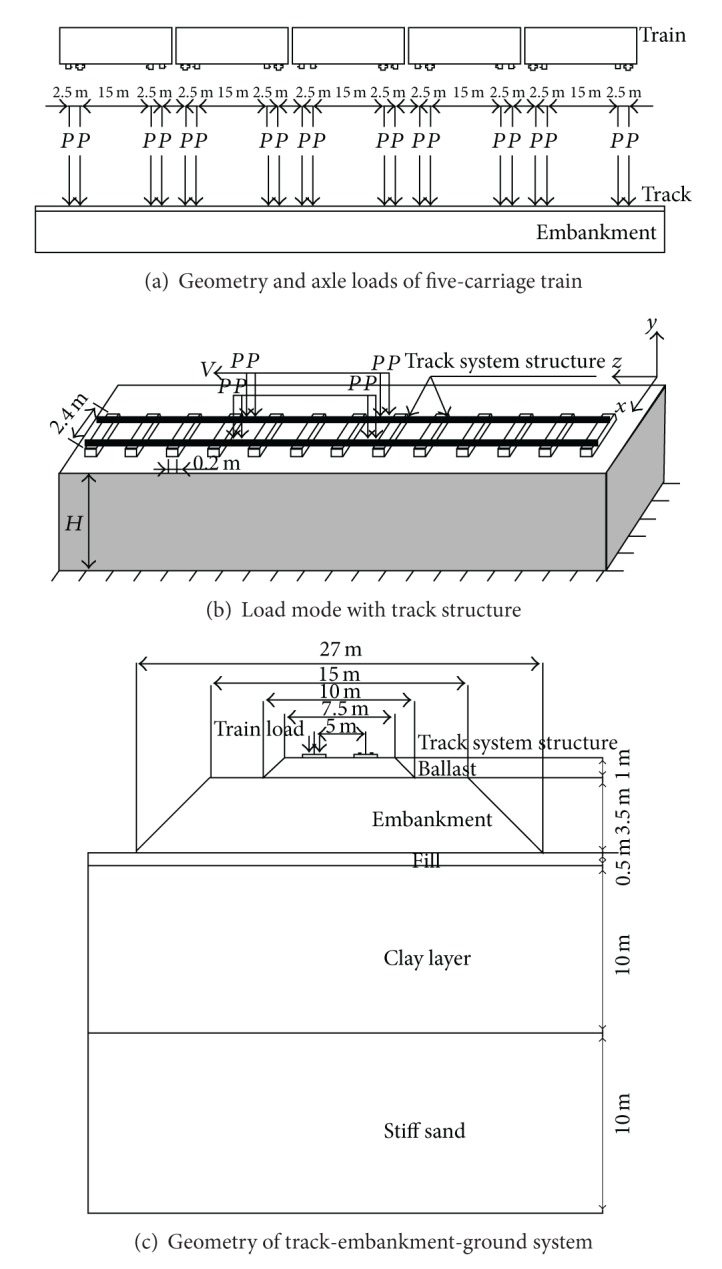
Load model and geometry of track-embankment-ground system.

The train axle load and geometry were shown in Figure 1. For the three-dimensional models, the elements in the rail are referred to as loading unit, and the moving unit loads were applied on the 8-noded solid elements simulating the rail. In this case, the load distribution *P* from the rail was calculated from the static solution of train axle load *F* acting on the element Δ*S*, the *P* is given by

(1) $$\begin{array}{c} P=\frac{F}{\Delta S}, \end{array}$$

where, *F* is 70 KN and Δ*S* is 0.02 m^2^, so the calculated distribution load *P* is 3.75 MPa. The load *P* was then applied directly on the rail elements with time shifts corresponding to the train speed. All these loading models generate stress waves just from the main source, that is, the track structure response. Other sources such as rail defects, unsteady riding of the vehicle, and variable support were not considered. All the finite element analyses in this project were performed in the time domain using direct time integration. The time step of the analyses was set to be automatic and corresponded with vibration characteristic of the whole system.

### 2.2. Finite Element Model

**Figure 2:**
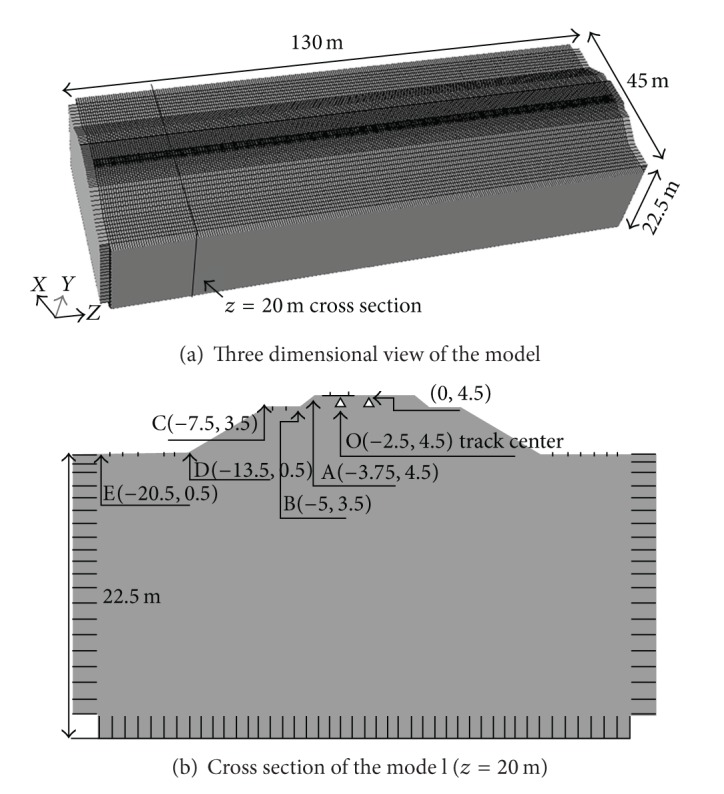
Finite element mesh of the three-dimensional model.

The model was set to 130 m in length. This was assumed as an initial estimate since only the response close to the track was considered. The three-dimensional model, consisted of rails, sleepers, ballast, and embankment, measured 45 × 130 × 27 m^3^, and consisted of 306,668 elements, is represented by 8-noded brick elements (see Figure 2). In the ballast and embankment, the average element body size was about 0.375 × 0.5 × 0.25 m. The rail and sleeper elements size was 0.05 × 0.2 × 0.05 m and 0.1 × 0.1 × 0.15 m, respectively. The whole system is divided into four single formulated substructures, that is, the track, ballast, embankment, and the ground, respectively. Ballast is placed on the top of embankment, supporting the rails and the sleepers. The fixed boundary was used in the bottom of the model. Infinite elements based on the previous work by Lysmer and Kuhlemeyer [41], and Kouroussis et al. [42] are used on the *X* and *Z* direction boundaries to represent the infinite boundary condition to absorb *S* and *P* waves. The nodes at the bottom boundary were fixed in every direction to simulate the bedrock. Both ends of the ground boundary were fixed in every direction in order to keep the ground in place at the ends of the finite element model. The explicit central difference method [43] is used for time integration of the dynamic equilibrium equations. Program ABAQUS (Dassault Systems 2007) is used for finite element simulation, in which both the Lysmer-Kuhlemeyer (LK) infinite element and the explicit solver are available integrated.

A parametric analysis is carried on considering different rail, ballast, embankment, and ground soil properties. The calculated parameters of finite simulation were shown in Table 1. The track, ballast, embankment, and ground are assumed to be linear elastic. Rayleigh damping represents energy-dissipating mechanisms in the ground. The mass and stiffness proportional damping constants of ground are *α* = 1.2 and *β* = 0.0004, which provide slight damping ratios of 2–4% in the frequency range of 3–50 Hz. Some observation points shown in Figure 2(b) are selected for further study.

## 3. Dynamic Responses of Track-Embankment-Ground System 

In order to investigate the dynamic characteristics of the track-embankment-ground system, the dynamic response due to five carriages travelling at different speeds was analyzed.

### 3.1. Dynamic Response of Track Structure

**Figure 3:**
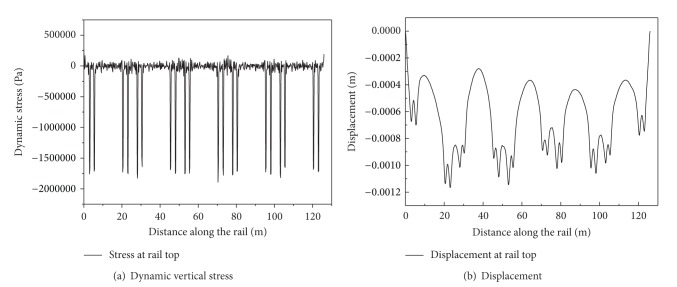
Dynamic responses along the rail top at time 1.54 s for five carriages travelling at a speed of 80 m/s.

Figure 3 shows dynamic responses along the rail at time 1.54 s for five carriages travelling at a speed of 80 m/s. The Figure shows that the peaks of the vertical stress along the rail appear at the position where the train load is applied, the values of which are around 1.7 MPa. The peaks of vertical displacement roughly coincide with the instantaneous position of the moving loading unit, of which the value is around 0.00117 m.

**Figure 4:**
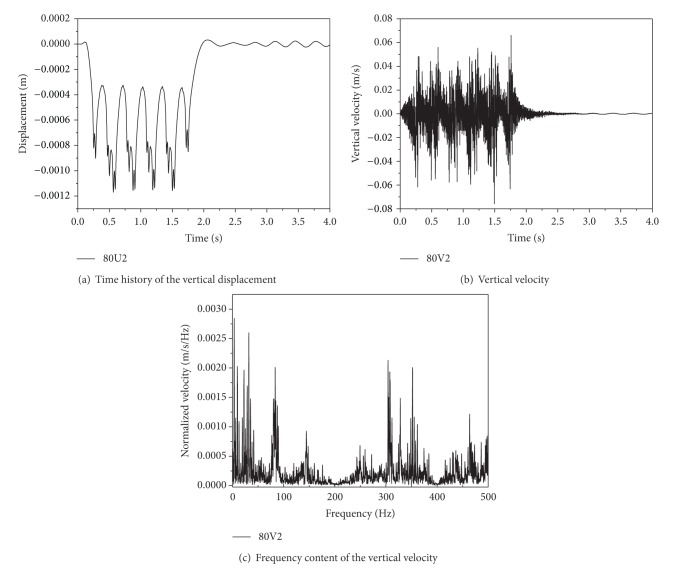
Curves of dynamic response at the rail top for five carriages travelling at *v* = 80 m/s (288 km/h).

Figure 4 shows the time history of the vertical displacement, velocity, and the frequency content of the vertical velocity at the rail for five carriages travelling at *v* = 80 m/s (288 km/h). In Figure 4(a), it can be seen that the peaks of vertical displacement at rail appear as the train loads passing by correspondingly. The maximum displacement induced by the following carriage of train loads is 0.00117 m, which is larger than that induced by the first sets of loads for the reason of dynamic superposition effect of moving loads. In Figure 4(b), the maximum value of vertical velocity at the rail surface is 0.075 m/s, which decreases as the train load goes away. As the interbogie spacing *L*_*b*_ = 2.5 m, the axle spacing *L*_*a*_ = 25 m, for speeds *v* = 80 m/s. The bogie passing frequency *f*_*b*_ = *v*/*L*_*b*_ = 3.2 Hz (low frequency) and the axle load passage frequency *f*_*a*_ = *v*/*L*_*a*_ = 32 Hz. In Figure 4(c), the frequency content of the vertical velocity at the rail shows peaks both in the bogie passing frequency 3.2 Hz and the axle passing frequency 32 Hz. The peak velocity appears in the low, medium, and high frequency area. Therefore, the rail response shows the contributions of the resonance frequency of the track model on the dynamic response of track-ballast-embankment-ground system. Similar results are presented by Kaewunruen and Remennikov [39]. The measured time history and frequency content of velocity at the sleeper during the passage of an AVE-Alstom HST travelling at a speed, *v* = 298 km/h on Track1, show peaks in the bogie passing frequency, *f*_*b*_ = *v*/*L*_*b*_ = 4.43 Hz, and related high-order harmonic frequencies and in the axle passing frequency, *f*_*a*_ = *v*/*L*_*a*_ = 27.59 Hz, as well.

### 3.2. Dynamic Response of Embankment-Ground System

**Figure 5:**
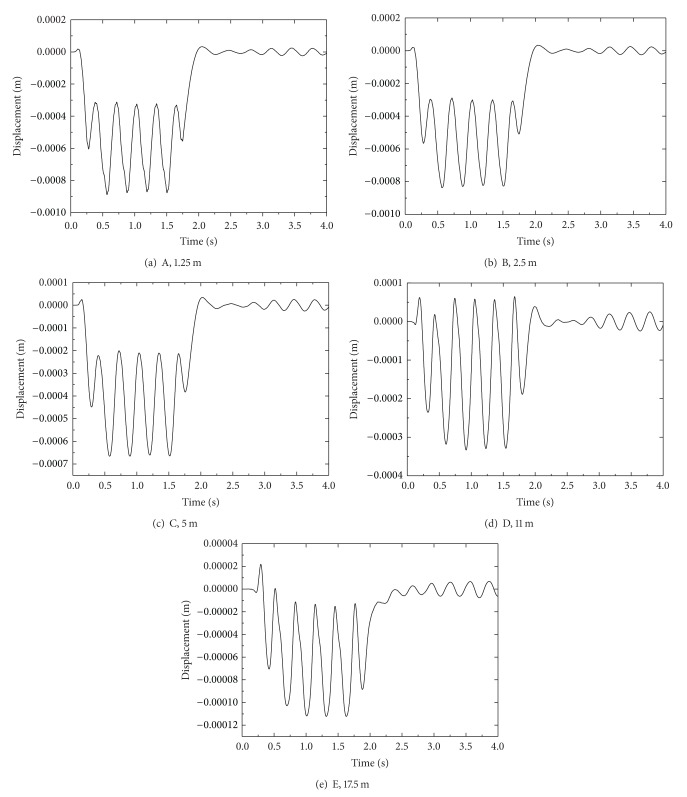
The time history of the vertical displacement at selected points at different distances from the track center.

The time histories of the vertical displacement at selected points A, B, C, D, and E (see Figure 2) at different distances from the track center are shown in Figure 5. It is noted that the vertical displacement at rail is larger than that at embankment, and the displacement peaks coincide with instantaneous position of the train load. However, the displacement peaks at other points appear to gradually get more out of phase and decrease as the distance from the track center increases. Thus, the vibration waves spread out from the track center and decrease for the vibration attenuation, which shows that the three-dimensional model is suitable for simulating the dynamic response of track-embankment-ground system.

**Figure 6:**
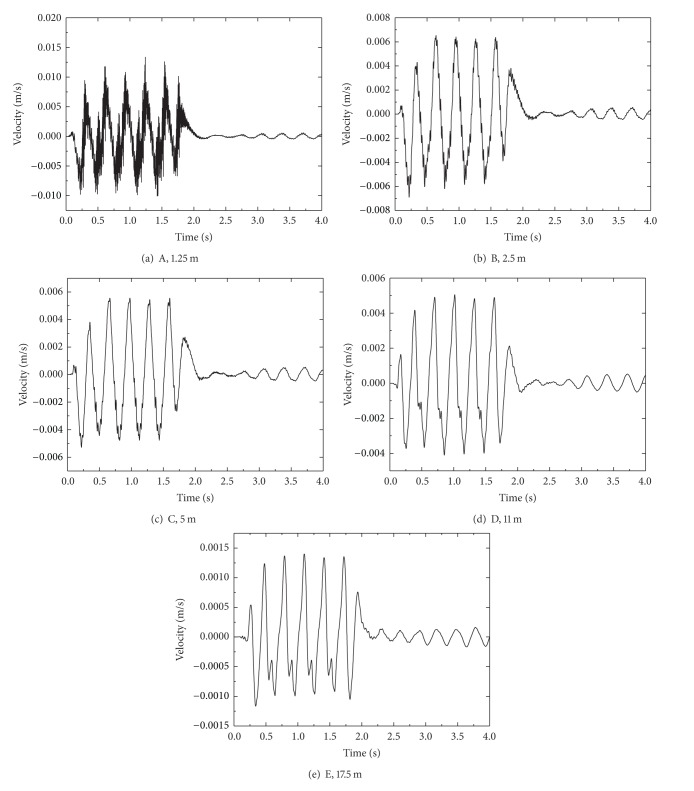
The time history of the vertical velocity at selected points at different distances from the track center.

**Figure 7:**
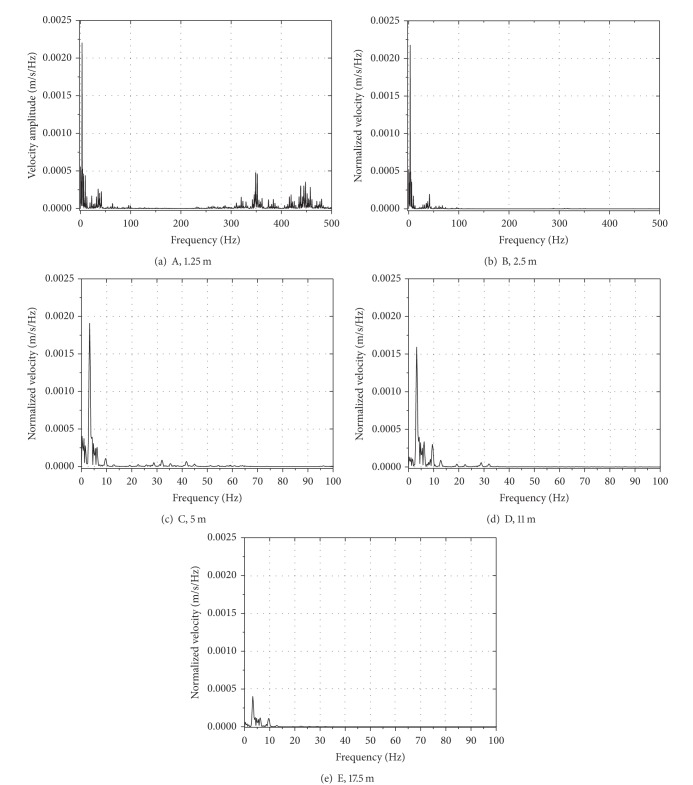
The frequency contents of the vertical velocity at selected points at different distances from the track center: *v* = 80 m/s.

Figure 6 shows the time histories of vertical velocity at selected points for train travelling with a speed of 80 m/s. It can be seen from the figure that with increase of distance to track center the velocity level of embankment and ground decreases gradually. The peak value of velocity at point A is 0.013 m/s, which decreases gradually with the increase of distance. It reaches 0.0013 m/s at 17.5 m. As mentioned previously, the dynamic response attenuations quickly within 17.5 m. According to the spectrum of velocity response in Figure 7, the peak amplitude points at different distances from the track center appear around different frequency areas, and the peak values of velocity are also different. The spectrum of velocity response consisted of low, medium, and high frequencies, showing the vibration character and level near the excitation source (shown in Figure 7(a)), as is shown in Figures 7(c), 7(d), and 7(e). The vibration velocity amplitude decreases with the decrease of distance from the track center, and the peaks move toward low frequency, appearing at frequency below 10 Hz.

**Figure 8:**
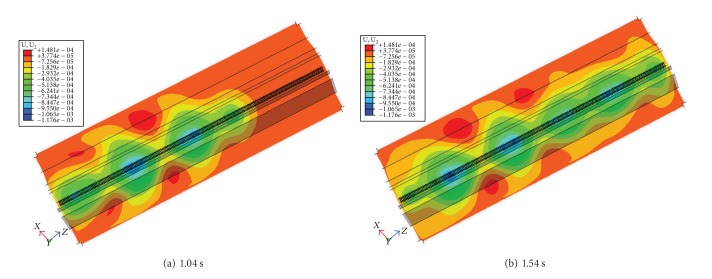
Typical contour plots of vertical displacement at different times for the speed *v* = 80 m/s.

Figure 8 shows the contour plots of the displacement for a train speed of 80 m/s at 1.04 s and 1.54 s. The moving loads enter the model at the left end and travel 130 m on the rail before leaving the model at the right end. The wave fronts of the induced stress waves were developed when they entered the finite element model. Instead, the wave fronts are created as soon as the moving loads, from which they originate, enter the model. The development of the wave fronts depends on the geometrical spreading of the waves and the propagation velocities of the different waves. It can here be observed that the wave fronts at the head consisted of circles at 1.04 s, and the circles induced by the latter loads spread out as the train passes by. The maximum downward displacements roughly coincide with the instantaneous position of the train loads. A dynamic superposition effect appears on ballast surface when the following wheel loads passing by, so the maximum displacement on the ballast surface for the first set of loads is lower than that for the following sets of loads. At the time of 1.54 s the wave fronts have become more developed and spread out from the source.

**Figure 9:**
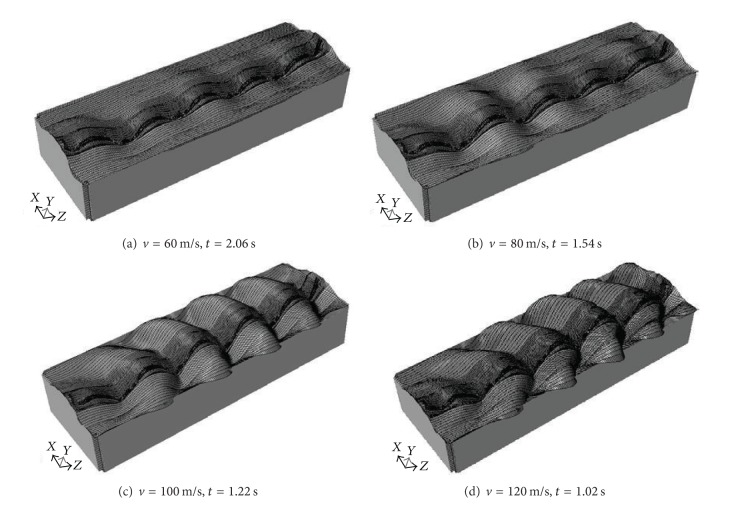
Full views of deformed meshes of displacement magnified with a factor of 15000 from finite analysis of train-induced ground vibrations for different train speeds travelling from left to right.

Here, for illustration purpose, the full views of deformed meshes of displacement for train speeds of 60 m/s, 80 m/s, 100 m/s, and 120 m/s are shown in Figure 9. For train speeds under subcritical ranges (*v* = 60 m/s) lower than the propagation velocity of the soft soil (71.6 m/s see Figure 9(a)), the induced wave fronts were perpendicular to the loads, and the deformation pattern is roughly symmetrical with each bogie imprint, which is similar to the displacement distribution for quasi-static case.

When the train speed is supercritical (see Figures 9(b), 9(c), and 9(d)), the induced wave fronts here show a plough-shaped behaviour following the loads, which are no longer perpendicular to the moving loads. Instead, they show plough shaped behaviour similar to the wash from a motor boat. The phase angles between the wave fronts and the loads moving direction decrease with the increase of moving speed. Behind the train, a series of trailing oscillations are observed. Similar results, in the case of three- dimensional model subjected to moving point loads, can be found in [34, 44]. Moreover, the motion orbits calculated from the three-dimensional analyses indicate that the Rayleigh wave is the dominating wave type outside the embankment [44]. At the critical speed case, the track critical velocity is slightly higher than the Rayleigh wave velocity due to the ballast acting as a beam [10]. Full Rayleigh wave effects therefore occur at speeds slightly higher than the Rayleigh ground wave speed.

**Figure 10:**
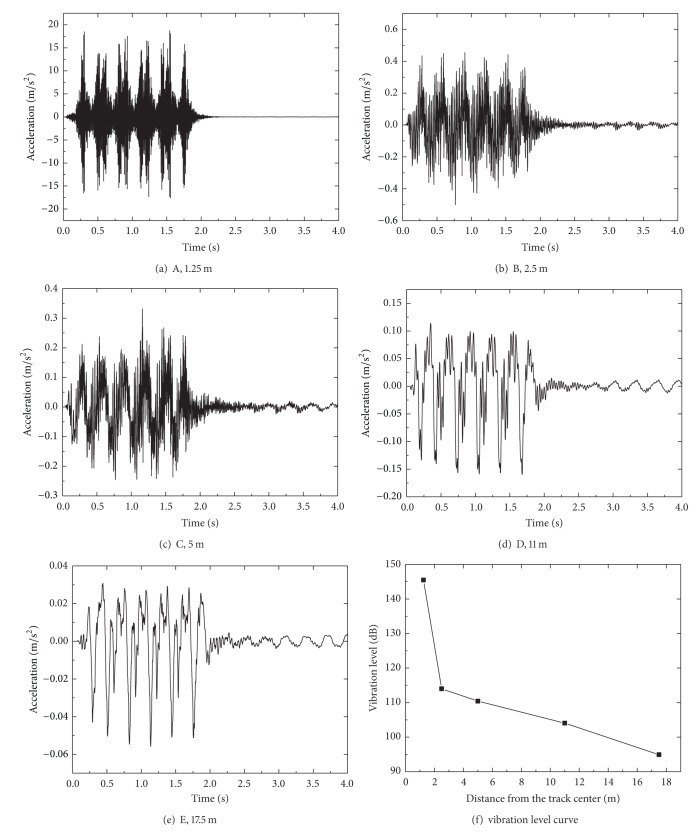
Time history of acceleration at selected points at different distances from the track center: *v* = 80 m/s ((a)–(e)) and (f) vibration level curve.

Time histories of acceleration at points A, B, C, D, and E and the vibration level curve are shown in Figure 10. The wheel effect and double peak appear in time history of vertical acceleration at distance 1.25 m from the track center under the train load. At this observation point near the excitation source, the maximum acceleration is approximately 18 m/s^2^. The acceleration amplitudes of the points located on the embankment and ground surface decrease with the increase of distance from the track center, which show attenuation according to the geometrical damping and internal damping of the soil. At a distance 17.5 m from the track center, the maximum acceleration is approximately 0.055 m/s^2^. The vibration measurements were taken out on embankment at Shinkansen in Japan, which show that the maximum acceleration appears around 5~20 m/s^2^. The vibration level limit in Japan is 90 dB. The formula [45] of acceleration vibration level adopted here is VAL = 20log⁡⁡(*a*_rms_/*a*_0_), where VAL is acceleration vibration level (dB), *a*_rms_ is effective value of vibration acceleration (m/s^2^), *a*_0_ is reference acceleration, and its value is 1*e* − 6 m/s^2^ according to ISO Standard [46]. The vibration levels of these points corresponding with distance from the track center are shown in Figure 8, decrease with the increase of distance from the track center. It can be seen that the vibration level at a distance 17.5 m is 94 dB close to the value limit 90 dB in Japan's vibration standard.

## 4. Parametric Study

Due to high speed train passage, track-ballast-embankment-ground properties play an important role in the ground vibration. Their effects can be evaluated by representing the actual ballast and embankment properties using the FEM presented in this paper. In order to investigate this effect, some different numerical simulations are carried out. The effects of train axle load and train speed are also discussed in the following section.

### 4.1. Effect of Train Speed

**Figure 11:**
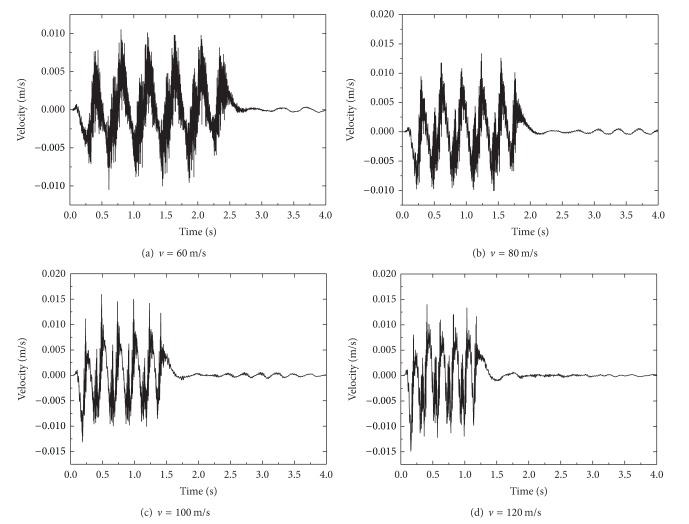
Time history of vertical velocity at a point 1.25 m from the track center for different train speeds.

This section conducts a preliminary study of the train load fundamental passing frequency effect on track-ballast-embankment-ground vibration under four train speeds (60 m/s, 80 m/s, 100 m/s, and 120 m/s, resp.). Since the ground Rayleigh wave speed is *C*_*R*_ = 71.6 m/s, these speeds are in the subcritical, critical, and supercritical ranges, respectively.  Figure 11 shows the time history of vertical velocity at a point 1.25 m from the track center for above train speeds. By referring to the time history, one can observe the passing of every bogie and axle of the train. It is noted that the vibration amplitude of vertical velocity increases with the train speed but tends to fall when the train speed (for *v* = 120 m/s see Figure 11(d)) comes near the fill Rayleigh wave speed *C*_*R*_ = 128.6 m/s.

**Figure 12:**
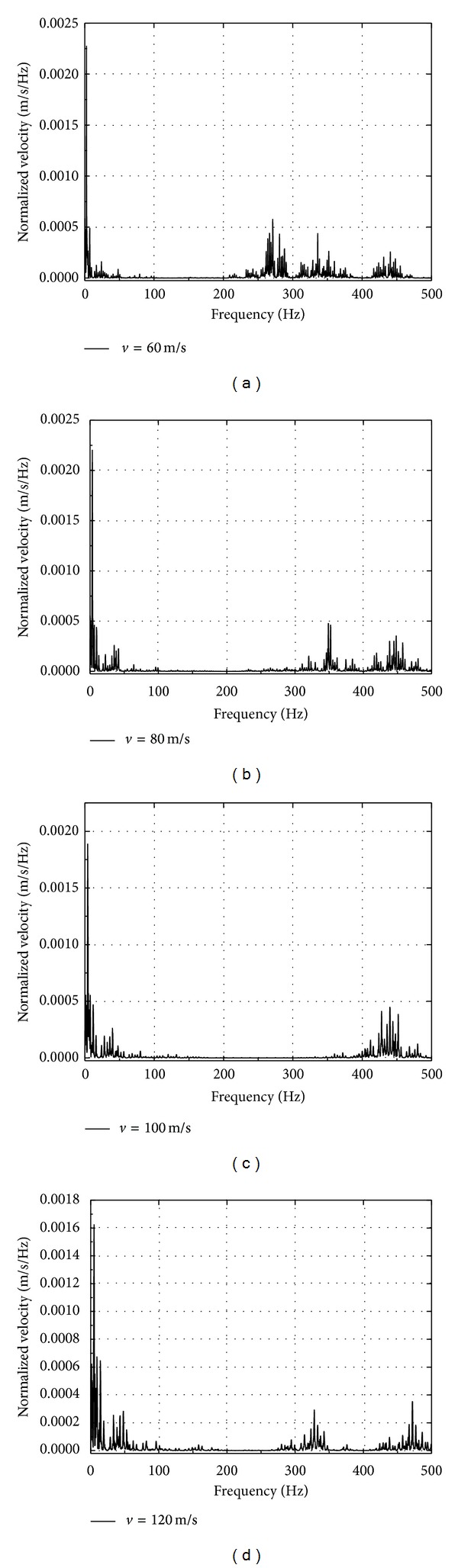
Frequency contents of vertical velocity at point 1.25 m from the track center for different train speeds.

Frequency spectra for the vertical velocity are obtained using the Fourier transform. Frequency spectra are shown in Figure 12 at point 1.25 m from the track center for different speeds. As the interbogie spacing *L*_*b*_ = 2.5 m, the axle spacing *L*_*a*_ = 25 m, and the bogie passing frequency *f*_*b*_ = *v*/*L*_*b*_, the axle load passage frequency *f*_*a*_ = *v*/*L*_*a*_. It can be observed from these figures that the frequency contents show peaks in the bogie passing frequency 2.4 Hz, 3.2 Hz, 4 Hz, and 4.8 Hz (low frequency) and in the axle load passage frequency 24 Hz, 32 Hz, 40 Hz, and 48 Hz for speeds mentioned above. Also peaks are noted that are associated with rail and sleeper vibration character in the high frequency range larger than 200 Hz. The velocity peaks tend to move toward high frequency range with the increase of train speeds, close to 450 Hz for train speed 80 m/s in Figure 12(b).

**Figure 13:**
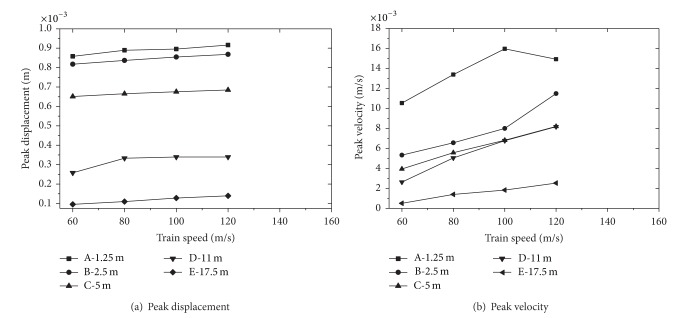
Peak displacement and velocity at selected points for different train speeds.

Figure 13 shows the effect of train speed on peak displacement and velocity on the ballast-embankment-ground surface. Values of the peak displacement at a distance from the track center for different speeds are shown in Figure 13(a). The peak at observation point decrease with the distance from the track center for speeds 60 m/s, 80 m/s, 100 m/s, and 120 m/s. For the selected points, the peak displacement increases with the increasing of train speed, but the amplitude is not obvious. Thus, the following conclusion can be obtained. The train speed has no significant effect on peak displacement.

Values of the peak velocity at selected points for different speeds are shown in Figure 13(b). It can be observed that the peak velocity increases with train speed for different points. The peak velocity depends significantly on the distance from the track center, decreases quickly with distance up to 5 m, and slows down from 5 m to 11 m, then decreases quickly again. The velocity attenuation tendencies in ballast, embankment, and ground are different due to the dynamic character of them.

### 4.2. Effect of Ballast Modulus

**Figure 14:**
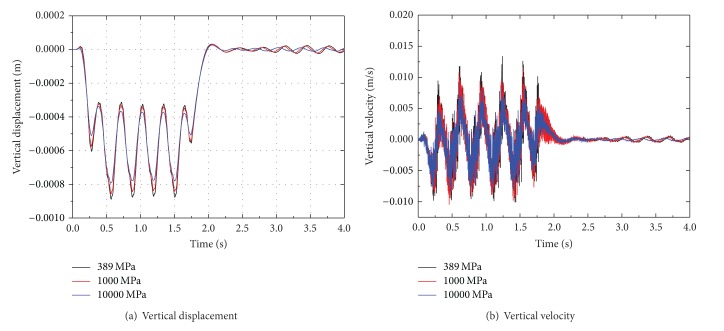
Time histories of dynamic response at point 1.25 m from the track center for different ballast modulus.

The dynamic response of ballast-embankment-ground is analyzed based on Young's modulus changes of ballast. Time histories of vertical displacement and velocity at point 1.25 m from the track center for different ballast modulus are shown in Figure 14. The vertical displacement and velocity both decrease with the increase of modulus. Thus, stiffening of ballast can reduce the vibration level and be realized by either installing a concrete slab under the sleeper or replacing the ballast with material with higher stiffness.

**Figure 15:**
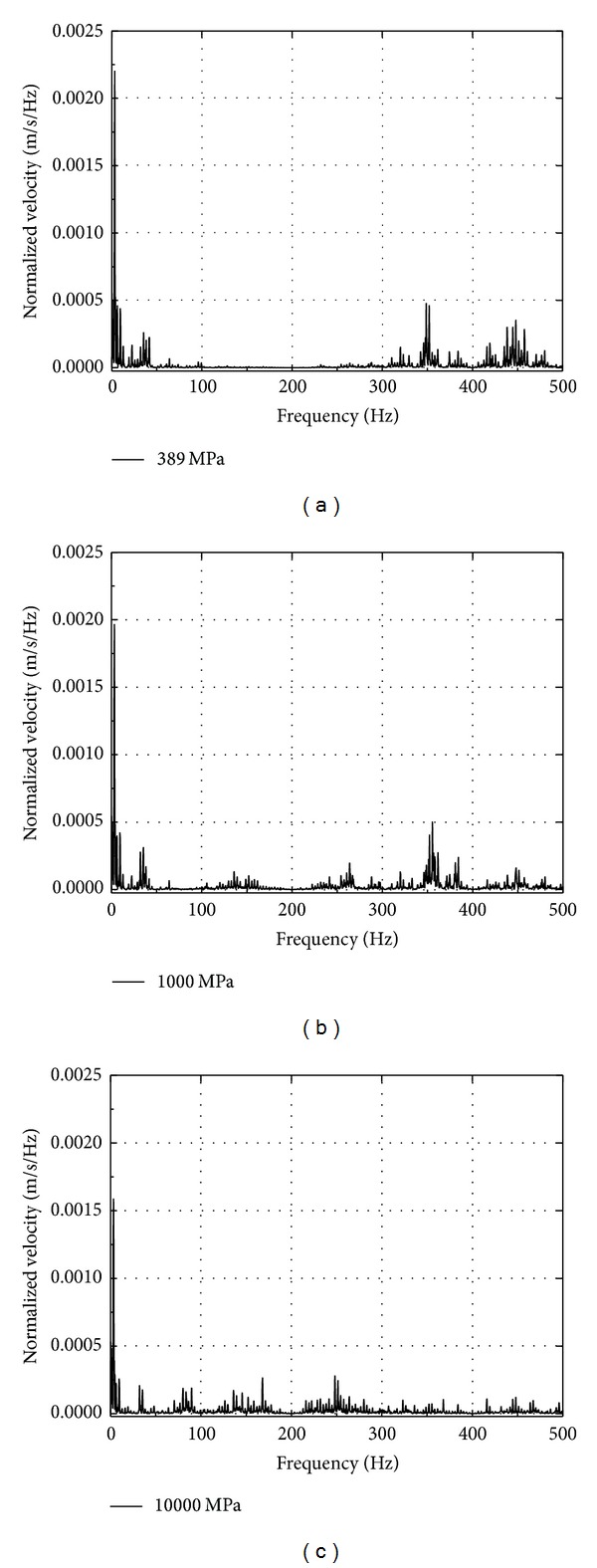
The velocity amplitude of Fourier spectrum for different ballast modulus.

The velocity amplitude of Fourier spectrum for different Young's modulus of ballast is shown in Figure 15. It is noted that the velocity amplitude that appears in low, medium, and high frequency ranges decreases with modulus of ballast and tends to move toward high frequency ranges. The peak velocity decreases with the decreasing of modulus, for example, at the value of 10000 MPa, moves back to medium frequency ranges, and appears around 80 Hz, 140 Hz, 170 Hz, and 250 Hz.

**Figure 16:**
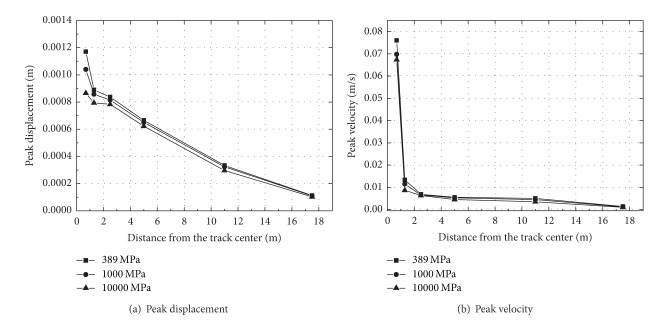
The peak displacement and velocity at a distance from the track center for different ballast modulus.

The peak displacement and velocity at a distance from the track center are shown in Figure 16. It can be observed from this figure that peak displacement and velocity decrease with the increasing of distance, especially at distances from 1.25 to 2.5 m away the track center. For example, the peak displacement at location 1.25 m decreases with the modulus of ballast, but this tendency vanished gradually with the distance away the track center. Similar results can be obtained for the peak velocity with the changing of ballast modulus. For ballast modulus is equal to 389 MPa, the peak velocity at the rail is 0.076 m/s more larger than that at point on ballast 1.25 m away the track center, of which the value is 0.013 m/s.

### 4.3. Effect of Fill Modulus

**Figure 17:**
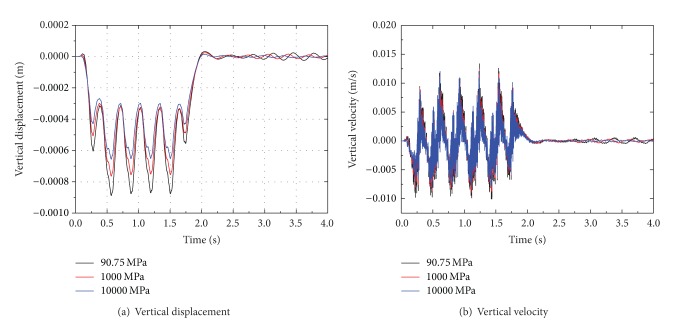
Time histories of vertical displacement and velocity at point 1.25 m from the track center for different fill modulus.

**Figure 18:**
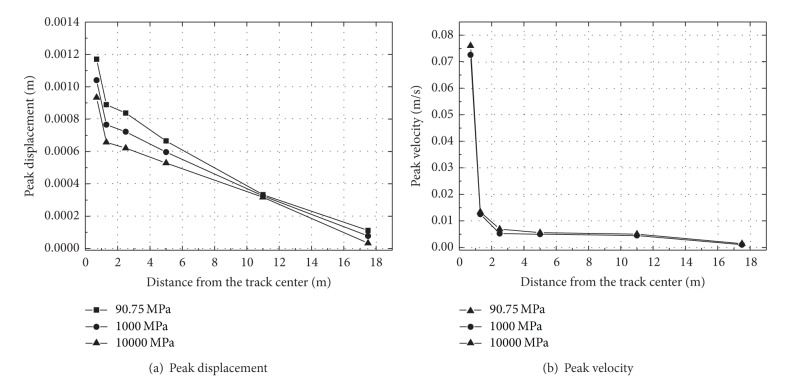
The peak displacement and velocity at a distance from the track center for different fill modulus.

To instigate the influence of fill, three moduli 90.75, 1000, and 10000 MPa are chosen. The dynamic response of ballast-embankment-ground is analyzed based on Young's modulus variance of fill under the embankment. Time histories of vertical displacement and velocity at point 1.25 m from the track center are shown in Figure 17. The vertical displacement and velocity both decrease with the increasing of fill modulus. The high value modulus (10000 MPa) may reduce the peak displacement by about 26% compared to the low value (90.75 MPa). The peak displacement and velocity at a distance from the track center are shown in Figure 18. A reduction of about 26% for the high-stiffness fill and 15% for medium-stiffness fill is achieved at point near the track center. But the influence of modulus on peak velocity is not obvious, as seen in Figure 18(b). So stiffening of fill under the embankment can reduce the vibration level, on the other hand, it can be realized by installing a concrete slab under the embankment. Moreover, improvement of soft soil ground is another method to reduce the vibration, which can be achieved by a variety of techniques, such as use of lime-cement piles [47].

**Figure 19:**
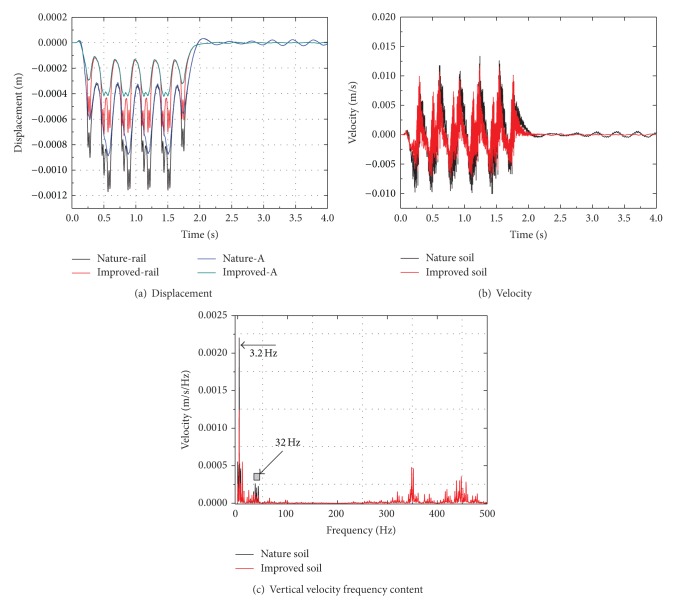
Dynamic response at rail and point A for natural and improved soil.

Figure 19 shows the effect of improvement of soft soil ground on the vibration produced in the system. In Figure 19(a), the maximum displacement at the rail reduces by about 40% with the improvement of soft soil, and the maximum displacement at point A 1.25 m from the track center reduces by about 55%. In Figure 19(b), the velocity decreases with the improvement of soft soil in accordance.  Figure 19(c) shows that the peak velocity decreases with the improvement of soft soil in low frequencies, mainly in the bogie passing frequency 3.2 Hz and the axle load passage frequency 32 Hz and tends to move from 32 to 3.2 Hz.

### 4.4. Effect of Axle Load

**Figure 20:**
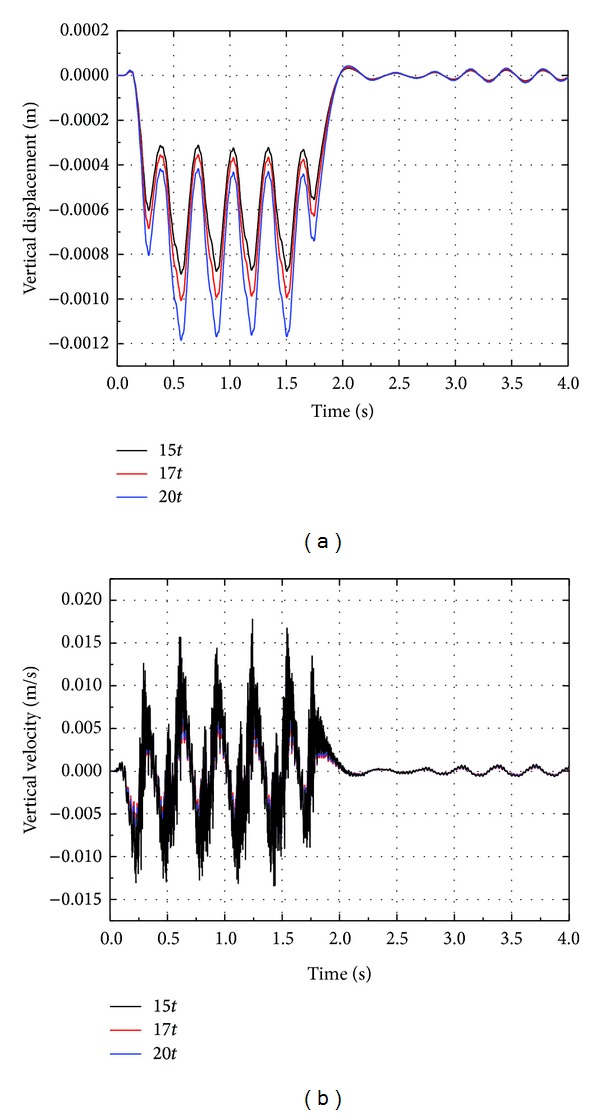
Time histories of vertical displacement and velocity at point 1.25 m from the track center for different axle loads.

Time histories of vertical displacement and velocity at point 1.25 m from the track center for different axle loads are shown in Figure 20. It can be noted that the vertical displacement and velocity increase with the axle load of high speed train. Compared to axle load 15*t*, the vertical displacement and velocity for axle load 20*t* increase by about 25%. Thus, the influence of axle load on the vibration of track-embankment-ground system is obviously lower than that of train speed.

**Figure 21:**
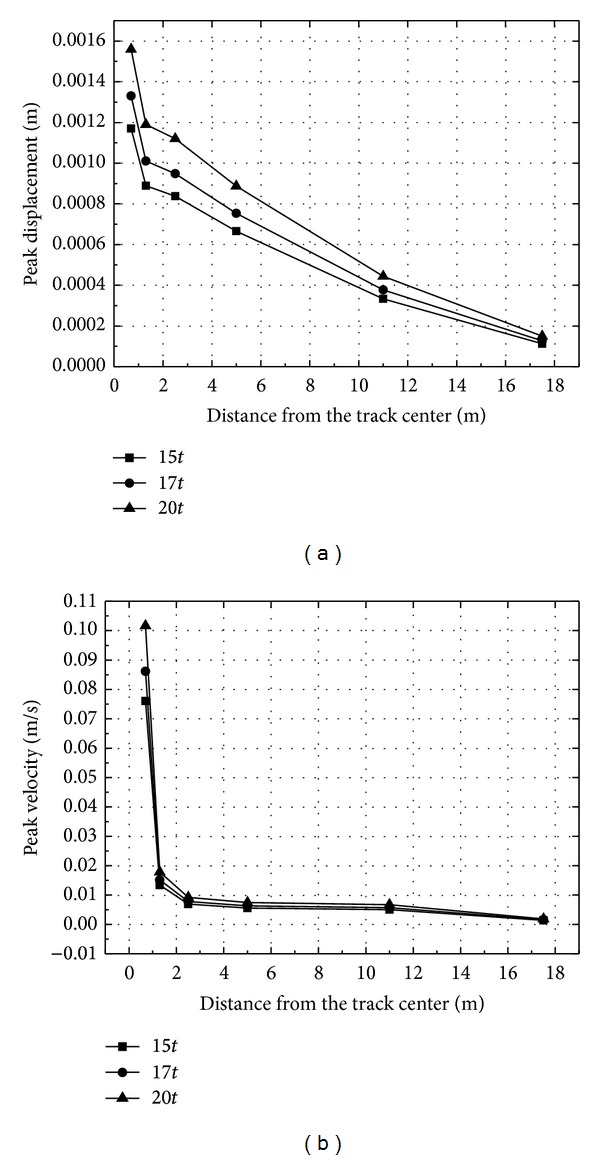
The peak displacement and velocity at a distance from the track center for different axle loads.

Figure 21 shows the peak displacement and velocity at a distance from the track center for different axle loads. The peak displacement decreases with the decreasing of the distance from the track center. The changing law of peak velocity is the same. In Figure 21(b), for the case of axle load 20*t*, the peak velocity at rail is 0.1 m/s, and the value of which is 0.018 m/s at point 1.25 m from the track center. The influence of axle load on peak displacement and velocity drops gradually with the decreasing of distance. It can be observed that the effective range of vibration from the track center is approximately 2.5 m. The vibration level is low when the dynamic waves reach the embankment.

## 5. Conclusions

In this paper, the finite element analyses of train-induced vibrations presented in this work demonstrate the feasibility of simulating and analyzing the response of track-embankment-ground system. The modeling procedure dealt with the geometric complexities induced by the shape of rail, sleeper, ballast, embankment, and the infinite extension of the natural layered ground. Moreover, the presented numerical model can predict the system vibrations induced by the passing of high speed trains, formulate design recommendations for structures near the tracks, identify the causes of high vibration levels, and evaluate settlements to attenuate them.

The results presented have shown that the numerical model describes the physical phenomenon with excellent accuracy. The fundamental components of the frequency content of the response are axle and bogie passing frequency, high-order harmonic frequencies, and the sleeper passing frequency. Also it is noted that the peaks associated with rail and sleeper vibration character in the high frequency range are larger than 200 Hz. The peaks of velocity tend to become high frequency values with the increasing of train speeds. The vibration amplitude of vertical velocity increases with the increasing of the train speed. The velocity attenuation tendencies in ballast, embankment and ground are different due to the dynamic character of them. Stiffening of fill under the embankment can reduce the vibration level, on the other hand, it can be realized by installing a concrete slab under the embankment.

However, in this study, there are no direct validations of those predictions against practical measurements. Only some comparisons are taken out between the numerical results and existing experimental measurements. Further study is needed to investigate the difference between numerical results and measured results to more accurately predict vibrations induced by high speed train loads.

## Figures and Tables

**Table 1:** Material parameters of finite simulation.

Material	*E*/Pa	*μ*	*ρ*/kg/m^3^	*V*_*S*_	*V*_*R*_
Ballast	3.89*E* + 8	0.3	2200	260.8	241.9
Embankment	2.5*E* + 8	0.3	1800	231.1	214.4
Fill	9.075*E* + 7	0.3	1800	138.7	128.6
Clay	25.36*E* + 86	0.35	1600	76.6	71.6
Stiff sand	1*E* + 8	0.3	1800	146.2	135.6
Rail	2.1*E* + 11	0.17	7800	3392	3074.2
Sleeper	2*E* + 10	0.2	2500	1826	1664.5

Note: *E* is Young's modulus; *ρ* is density; *μ* is Poisson's ratio; *V*_*S*_ and *V*_*R*_ are shear and Rayleigh wave speeds, respectively.
